# Exogenous Pulmonary Surfactant as a Vehicle for Antimicrobials: Assessment of Surfactant-Antibacterial Interactions *In Vitro*

**DOI:** 10.1155/2014/930318

**Published:** 2014-04-29

**Authors:** Alexei Birkun

**Affiliations:** Department of Emergency Medicine and Anesthesiology, Crimea State Medical University, Lenin Avenue 5/7, Simferopol 95006, Ukraine

## Abstract

Owing to its unique surface-active properties, an exogenous pulmonary surfactant may become a promising drug delivery agent, in particular, acting as a vehicle for antibiotics in topical treatment of pneumonia. The purpose of this study was to assess a mutual influence of natural surfactant preparation and three antibiotics (amikacin, cefepime, and colistimethate sodium) *in vitro* and to identify appropriate combination(s) for subsequent *in vivo* investigations of experimental surfactant/antibiotic mixtures. Influence of antibiotics on surface-active properties of exogenous surfactant was assessed using the modified Pattle method. Effects of exogenous surfactant on antibacterial activity of antimicrobials against *Staphylococcus aureus*, *Klebsiella pneumoniae*, and *Pseudomonas aeruginosa* were evaluated using conventional microbiologic procedures. Addition of amikacin or cefepime to surfactant had no significant influence on surface-active properties of the latter. Obvious reduction of surface-active properties was confirmed for surfactant/colistimethate composition. When suspended with antibiotics, surfactant either had no impact on their antimicrobial activity (amikacin) or exerted mild to moderate influence (reduction of cefepime bactericidal activity and increase of colistimethate bacteriostatic activity against *S. aureus* and *P. aeruginosa*). Considering favorable compatibility profile, the surfactant/amikacin combination is advisable for subsequent investigation of joint surfactant/antibacterial therapy in animals with bacterial pneumonia.

## 1. Introduction

The success of pneumonia treatment in large part is defined by effectiveness of antimicrobial therapy. In turn, efficiency of antibacterial therapy depends on formation of sufficient antimicrobial drug concentration in the site of infection [1]. Direct administration of antibiotics into a tracheobronchial tree using nebulization or intratracheal instillations can provide high concentration of drug in a focus of infection with low level of systemic absorption and, therefore, decreased systemic toxicity [2]. However, in patients with pulmonary diseases directly administered drug may predominantly accumulate in central rather than in peripheral airways. This negative effect is attributable to physiologic properties of fluid distribution in respiratory pathways and to specific inflammatory alterations such as bronchial hypersecretion, bronchoconstriction, and bronchial edema [3–5].

It was hypothesized that use of exogenous pulmonary surfactant as a vehicle for antibacterial agents could enhance the efficiency of local antimicrobial therapy in pneumonia [6, 7]. Pulmonary surfactant is a lipoprotein complex produced by cells of respiratory tissue that ensures a variety of essential physiologic functions [8]. One of the most important features of surfactant is the ability to decrease a surface tension, thus providing normal alveolar ventilation and gas exchange and preventing alveolar collapse [9]. It has been shown that, owing to its specific composition, an exogenous surfactant promotes effective and uniform peripheral distribution of fluid in lungs [6]. Furthermore, given the fact that exogenous pulmonary surfactant is able to reexpand atelectatic areas, it is anticipated that, after local administration with surfactant, antimicrobials will deposit in effective concentrations even in collapsed areas of lungs which are more likely to be infected [5].

Despite the theoretical feasibility for use of exogenous surfactant as a vehicle for antimicrobials, this method is still not realized in clinical settings. Scientific achievements in this area are limited to several preclinical studies [6, 7, 10–13]. These studies have shown that interactions between exogenous surfactant and antibiotics are possible. Therefore, alterations in activity of both substances should be considered and thoroughly evaluated before the clinical use of any surfactant-antibiotic mixtures [10].

The purpose of this study was to evaluate potential interactions between exogenous surfactant and three antibiotics (amikacin, cefepime, and colistimethate sodium)* in vitro* and, consequently, to determine a suitable combination(s) for subsequent investigations of surfactant/antibiotic experimental mixtures intended for local treatment of pneumonia. The study consisted of two parts: (1) assessment of influence of the antibiotics on surface-active properties of the exogenous surfactant and (2) assessment of influence of the exogenous surfactant on antibacterial properties of the antimicrobials.

## 2. Materials and Methods

### 2.1. Surfactant and Antibiotics

Sterile natural pulmonary surfactant preparation (Suzacrin, Docpharm, Ukraine) was used. This emulsion includes purified phospholipids (76%; 50 mg per 1 mL of emulsion) isolated from porcine lung homogenates and physiological solution as an excipient. Suzacrin contains surfactant-associated proteins B and C but is free of surfactant-proteins A and D.

The following antibiotics were utilized: amikacin (Amicil, Kievmedpreparat, Ukraine), cefepime (Cefepime, Nectar Lifesciences Ltd., India), and colistimethate sodium (Colomycin Injection, Forest Laboratories UK Ltd., UK). These antibacterial agents are currently recommended to treat pulmonary infections, caused by multidrug-resistant pathogens.

Solutions of antibiotics, emulsion of surfactant, and experimental mixtures of antibiotics in surfactant were freshly prepared in aseptic conditions using sterile 0.9% NaCl solution immediately before testing.

Specific requirements were established to standardize experiments and to provide a balanced calculation of antibiotic and surfactant amount in experimental mixtures: quantitative ratio of antibiotic and surfactant should correspond to the ratio of mean daily doses recommended for adult male patient with a body mass of 70 kg, that is, surfactant phospholipids—700 mg, cefepime—2,000 mg, amikacin—1,000 mg, and colistimethate sodium—4,000,000 IU (320 mg).

### 2.2. Test Strains and Culture Media

The following test strains were provided by Crimean Sanitary-Epidemiological Station (Simferopol, Ukraine):* Staphylococcus aureus* ATCC25923,* Klebsiella pneumoniae* 3534/51, and* Pseudomonas aeruginosa* ATCC27853. Hottinger broth (Federal State Institution of Science “State Research Center of Applied Microbiology and Biotechnology,” Obolensk, Russia; amine nitrogen concentration—132 mg%) and meat-peptone agar (MPA; State Pilot Factory of Bacterial Cultures of the Institute of Milk and Meat Technology, Kiev, Ukraine; amine nitrogen concentration—2.7 mg%) were used as culture media to investigate susceptibility of bacteria to antibiotics, surfactant, and surfactant-antimicrobial mixtures.

### 2.3. Assessment of Surface-Active Properties

The commercial surfactant preparation was diluted (reemulsified) in sterile 0.9% NaCl solution (control) or similar volume of antibiotic solution (three test series) to produce equal final phospholipid concentration of 20.6 mg/mL. The total volume of prepared experimental mixtures was 34 mL (a volume allowable for intratracheal administration to adult person within 24 hours). Final concentrations of antibiotics in surfactant-containing mixtures were as follows: amikacin—29.4 mg/mL, colistimethate sodium—117,647 IU/mL, and cefepime—58,8 mg/mL.

Apart from the experimental series, five subsidiary series were investigated: undiluted surfactant preparation (phospholipid concentration—50 mg/mL), solutions of amikacin, colistimethate sodium, and cefepime in concentrations recommended for clinical use (167 mg/mL, 500,000 IU/mL and 100 mg/mL, resp.), and diluent (0.9% NaCl solution).

The experiment was conducted at a temperature of 26°C. Test suspensions, as well as control and subsidiary samples (five samples for every series), were foamed in sterile tubes with single-use medical syringes equipped with 23G injection needles. Foam was formed by triple aspiration and return of test material into a tube. The modified Pattle method [14, 15] was utilized to assess surface-active properties in stable foam. This method enables an evaluation of surface tension force dynamics on a liquid-gas interphase boundary according to the changes of air bubble diameter. For this purpose, hanging drops of obtained foam were examined in transmitted light using Olympus CX41 microscope equipped with Olympus C5050Z digital camera. One field of vision (200× magnification) uniformly filled with bubbles was photographed in every drop in automatic mode during 20 min with an interval of 30 sec. Thereby, each replication in a series included 41 shots (205 shots for each series composed of 5 replications). Obtained images (a total of 1,230 images) were analyzed using Olympus DP-Soft 3.11 application. Changes of 20 randomly chosen bubbles were monitored through all images of every replication. Bubble diameter (*μ*m) was measured for the first and final (obtained after 20 min) images only. Totally, 1,200 bubble measurements were performed during this experiment. Stability index (SI) was calculated for each replication using the formula

(1) $$\begin{array}{c} \text{SI}=\frac{\sum d_{x}^{2}}{\sum d_{y}^{2}}, \end{array}$$

when *d*_*x*_ is a result of final measurement in a series (41st image) and *d*_*y*_ is a result of first measurement (1st image).

### 2.4. Susceptibility Testing

Minimum inhibitory concentrations (MIC) of test substances were determined using conventional technique of serial dilution in Hottinger broth. Each series of dilutions was investigated in two replications. The volume of liquid culture medium in each tube amounted to 1 mL. During the antimicrobial activity assessment of antibiotics and mixtures of surfactant with antibiotics the quantity of surfactant and antibacterials in the initial tubes of series corresponded to aforecited mean daily dose ratios. When assessing surfactant's inherent influence on the bacterial cultures, surfactant concentration in the initial tube of series was 5,000 mg/L. Test substances were introduced into the tubes in a volume of 0.170 mL (0.100 mL of amikacin and colistimethate sodium solutions and 0.070 mL of surfactant suspension) or 0.135 mL (0.100 mL of cefepime solution and 0.035 mL of surfactant suspension). Then, these tubes were filled up with Hottinger broth till the final volume of 1 mL. Maximum test concentration matched 1,000 mg/L for amikacin and cefepime, and 320 mg/L for colistimethate sodium. Maximum test concentration of surfactant in mixtures with antibiotics was 700 mg/L (for amikacin and colistimethate sodium) or 350 mg/L (for cefepime). Sequential twofold dilution of initial tube contents has been performed using the Hottinger broth. Then, the bacterial suspensions were introduced in all tubes in the volume of 0.1 mL. The bacterial suspensions were prepared from diurnal bacterial cultures using the same broth. The density of introduced bacterial suspension amounted to 5 × 10^4^ CFU/mL. Obtained mixtures were incubated within 24 hours at 37°C. Then, MIC was determined as a concentration of test substance in the last tube with clear broth in the presence of bacterial growth in control tube (free of test substances).

In order to determine a minimum bactericidal concentration (MBC; lowest concentration of substance that results in full inhibition of bacterial vital activity) 0.1 mL aliquots of test material from the tubes with transparent broth (with no visible bacterial growth) were transferred with a loop to MPA-containing plates. Subsequent to incubation within 24 hours at 37°C, MBC was determined as a minimum concentration of test substance that corresponded to the last plate in a series where microbial growth was completely absent.

### 2.5. Statistics

Most data are presented as a mean ± standard error. Data were compared using the unpaired Student's *t*-test. A probability level of *P* < 0.05 was considered to be statistically significant. Statistical analysis was performed using STATISTICA software version 10.0 (Stat Soft Inc., Tulsa, OK, USA).

## 3. Results

### 3.1. Assessment of Surface-Active Properties

The assessment of foaming properties of the officinal surfactant preparation, antibiotics (in therapeutic concentrations), and solvent demonstrated a wide range of surface activity characteristics (Table 1): from almost complete absence of foaming ability in normal saline to the formation of stable foam (which persisted ≥ 20 min) in surfactant and colistimethate. Foaming properties of amikacin and cefepime solutions were rated as intermediate, but close to normal saline.

Bubbles of colistimethate sodium solution were significantly larger than bubbles of surfactant suspension both in the beginning (*P* < 0.001) and at the end of the experiment (*P* < 0.001). The diameter of colistimethate sodium and surfactant bubbles decreased during the experiment by 21% and 8%, respectively (*P* < 0.001). SI values also suggested significantly higher surface activity of surfactant as compared with colistimethate sodium (*P* < 0.001).

Comparison of surfactant properties before and after dilution with normal saline (Tables 1 and 2) showed that 2.4-fold decrease of phospholipid concentration (20.6 mg/mL versus initial 50 mg/mL) does not result in significant changes of surface activity as per SI value (*P* > 0.05). However, the diameter of bubbles of diluted surfactant was significantly higher as compared with diameter of original surfactant suspension bubbles both in the beginning and at the end of the experiment (*P* < 0.001), thus suggesting a trend for reduction of surface activity.

As compared with control, experimental surfactant/amikacin mixture demonstrated smaller diameter of bubbles (Table 2) both in the beginning and at the end of the experiment (*P* < 0.001), as well as significantly lower tendency for reduction of bubble size in 20 min (*P* < 0.001), whereas there were no substantial changes of SI (*P* > 0.05).

The surfactant/cefepime mixture also retained high surface activity as per SI values (*P* > 0.05). However, this mixture differed from the control by greater bubble diameter in the beginning of the experiment (*P* < 0.01) and somewhat more pronounced, or accelerated, reduction of bubble size (*P* < 0.01).

There was no statistically significant difference in initial bubble size between surfactant/colistimethate sodium and control mixtures (*P* > 0.05), whereas experimental mixture bubbles had substantially smaller diameter than surfactant bubbles at the end of the experiment (*P* < 0.001). Therefore, a trend for bubble diameter decrease was more pronounced after antibiotic addition (*P* < 0.001). Relevant SI decrease was observed in this experiment as compared with control (*P* < 0.01), original surfactant preparation (*P* < 0.001), or colistimethate sodium solution (*P* < 0.05) (Table 2).

### 3.2. Susceptibility Testing

The exogenous surfactant preparation was devoid of apparent intrinsic antimicrobial effects as for* S. aureus*,* K. pneumoniae,* and* P. aeruginosa* cultures.

Amikacin demonstrated its efficiency against all three bacterial strains. This antibiotic completely inhibited growth of* S. aureus* and* P. aeruginosa *in 256-fold dilution, as well as growth of* K. pneumoniae*—in 526-fold dilution, that is, in concentration of 3.9 and 1.9 mg/L, respectively (Table 3). Antibacterial properties of amikacin were not affected when combined with surfactant suspension—MIC and MBC remained on the same level.

Cefepime exhibited most pronounced antistaphylococcal activity (MIC ≤ 0.49 mg/L, MBC = 1.9 mg/L). However, when tested against* P. aeruginosa*, MBC of cefepime was significantly (128-fold) higher than MBC of amikacin, whereas antipseudomonal MICs of these antibiotics were equally substantial.* K. pneumoniae* demonstrated full resistance to cefepime (bacterial growth was observed in all dilutions both in broth and after reinoculation on agar). Suspending of exogenous surfactant in cefepime resulted in slight decrease of bactericidal activity against* S. aureus* and* P. aeruginosa* cultures.

Colistimethate sodium had significantly lower antibacterial activity as compared to amikacin (against all three cultures) and cefepime (against* S. aureus*). The ability of colistimethate sodium to inhibit growth of* K. pneumoniae* and* P. aeruginosa *was moderate but higher than in cefepime. When combined with surfactant, bacteriostatic effect of colistimethate increased 4- and 2-fold for* S. aureus* and* P. aeruginosa*, respectively, though remaining on relatively low level (20 mg/L and 10 mg/L), while bactericidal activity was unchanged.

## 4. Discussion and Conclusions

It has been previously shown that interactions between antibiotics and surfactant may exist, and these interactions may influence efficiency of either substances [7, 10]. The purpose of this study was to assess potential mutual influence of natural surfactant preparation and three antibiotics (amikacin, cefepime, and colistimethate sodium)* in vitro* and to identify appropriate combination(s) for subsequent investigations of topical surfactant/antibiotic therapy in experimental acute bacterial pulmonary infection. Potential limitations of this study include restrictions as regards extrapolation of the results to other exogenous surfactant preparations due to differences in chemical composition of currently available surfactant products.

In the first part of the study it has been revealed that addition of the amikacin or cefepime to the surfactant had no significant influence on the surface-active properties of the latter, whereas obvious reduction of this feature was detected when mixing surfactant with colistimethate sodium. Considering the ability of colistimethate sodium to disrupt phospholipid bilayer of bacterial cell envelope [16], supposedly the observed effect may be caused by specific interaction of the colistimethate sodium with phospholipids of the surfactant.

The results of microbiological part of the study suggest that the surfactant preparation was devoid of intrinsic antibacterial activity. When mixed with antibiotics, the surfactant either had no impact on antimicrobial activity of the latter (e.g., amikacin entirely preserved its properties) or exerted mild to moderate influence. Specifically, there was a trend for decrease of cefepime bactericidal activity and increase of colistimethate sodium bacteriostatic activity against* S. aureus* and* P. aeruginosa*.

Interaction between exogenous surfactant and antibiotics was previously studied by van't Veen et al. [7, 10]. They have determined that surface-active properties of bovine pulmonary surfactant were not altered after addition of ceftazidime or pentamidine, whereas activity of surfactant was depressed with amphotericin B and amoxicillin. Surfactant surface-active properties in tobramycin/surfactant mixture were dependent on the type of solvent used. Surface-active properties of the surfactant were depressed when saline was utilized as a solvent but unaffected when tobramycin was mixed with surfactant in 0.2 M NaHCO_3_ buffer [10].

An ability of exogenous surfactant to suppress activity of certain antibiotics was demonstrated* in vitro* [7] in terms of tobramycin, whereas bactericidal activity of amoxicillin and ceftazidime was not altered. The authors have explained the depression of tobramycin antibacterial activity against* K. pneumoniae*,* P. aeruginosa*,* S. aureus,* and* Streptococcus pneumoniae* by the ability of aminoglycosides to bind negatively charged phospholipids contained in surfactant preparation and assumed that an excess of phospholipids can inactivate almost all the aminoglycosides [5, 7].

In contrast to the aforecited tobramycin/surfactant compatibility issues [7, 10], the results of present study indicate that activity of amikacin (another aminoglycoside antibiotic) was not suppressed by the surfactant preparation, and surface-active properties of the surfactant appeared unaffected when saline was used as a solvent for antibiotic, suggesting that not all aminoglycosides possess negative interaction with exogenous surfactant, at least considering specific features of given experiments (different surfactant preparation, different concentration of surfactant in the mixture, etc.).

Assuming relatively low activity of the colistimethate sodium against tested bacterial cultures, as well as noticeable reduction of surfactant surface activity in the surfactant/colistimethate sodium mixture, the use of this composition in subsequent studies in animals infected with* S. aureus*,* K. pneumoniae,* and* P. aeruginosa* appears to be unreasonable. Conversely, it is advisable to use the surfactant/amikacin composition for experimental studies in animals infected with given strains of* S. aureus*,* K. pneumoniae,* and* P. aeruginosa*, and the surfactant/cefepime composition for experimental staphylococcal infection in laboratory animals.

in summary, the study showed that an addition of therapeutic concentrations of the amikacin and cefepime to the exogenous surfactant preparation does not cause a decrease of surfactant surface activity* in vitro*, whereas significant suppression of both surfactant and colistimethate sodium surface-active properties is evident when mixed in one volume. Addition of the surfactant suspension to amikacin had no influence on high antimicrobial activity of the latter, whereas suspending surfactant with cefepime or colistimethate sodium resulted in a trend for decrease of cefepime bactericidal activity and increase of colistimethate sodium bacteriostatic activity against* S. aureus* and* P. aeruginosa*. Obtained data confirm the necessity of surfactant-antibacterial interaction assessment before utilization of any surfactant/antibiotic mixtures in clinical settings and in preclinical animal studies and show that further investigation of combined surfactant/antibacterial therapy should include an evaluation of the surfactant/amikacin composition in animals with bacterial pneumonia.

## Figures and Tables

**Table 1:** Foaming properties and stability index values for original preparations.

Foaming and surface activity characteristics	Surfactant suspension	Colistimethate sodium solution	Amikacin solution	Cefepime solution	NaCl solution
Foam properties	Stable, >20 min	Stable, >20 min	Unstable, 5–7 sec	Unstable, 10–15 sec	No foam
Diameter of bubbles, M ± m (*n*), *μ*m:					
(a) start of experiment	51.55 ± 1.06 (100)	110.62 ± 1.65 (100)	—	—	—
(b) end of experiment	47.46 ± 0.94 (100)	87.54 ± 2.27 (100)	—	—	—
Difference between (a) and (b)	4.09 ± 0.29 (100)	23.07 ± 1.58 (100)	—	—	—
Stability index (SI)	0.85 ± 0.02 (5)	0.65 ± 0.03 (5)	—	—	—

Note: M ± m: mean ± standard error.

**Table 2:** Surface activity parameters of experimental mixtures.

Surface activity characteristics	Surfactant + NaCl (control)	Surfactant + colistimethate sodium	Surfactant + amikacin	Surfactant + cefepime
Diameter of bubbles, M ± m (*n*), *μ*m:				
(a) start of experiment	77.98 ± 2.03 (100)	83.23 ± 3.23 (100)	56.04 ± 2.01 (100)	86.55 ± 2.17 (100)
(b) end of experiment	67.35 ± 1.56 (100)	53.52 ± 3.14 (100)	51.45 ± 2.12 (100)	70.20 ± 1.86 (100)
Difference between (a) and (b)	10.62 ± 1.18 (100)	29.71 ± 2.23 (100)	4.59 ± 0.62 (100)	16.34 ± 1.33 (100)
Stability index (SI)	0.76 ± 0.04 (5)	0.49 ± 0.06 (5)	0.85 ± 0.05 (5)	0.67 ± 0.04 (5)

Note: M ± m: mean ± standard error.

**Table 3:** Bacteriostatic and bactericidal characteristics of antibiotic solutions and experimental surfactant/antibacterial mixtures.

Antibiotics and antibiotic/surfactant compositions	MIC and MBC data (mg/L)					
	*S. aureus *		*K. pneumoniae *		*P. aeruginosa *	
	MIC	MBC	MIC	MBC	MIC	MBC
Amikacin	3.9	3.9	1.9	1.9	3.9	3.9
Amikacin + surfactant	3.9	3.9	1.9	1.9	3.9	3.9
Cefepime	≤0.49	1.9	>1,000	>1,000	3.9	500
Cefepime + surfactant	≤0.49	3.9	>1,000	>1,000	3.9 (1.9)	1,000
Colistimethate sodium	80	80	20	160	20	160
Colistimethate sodium + surfactant	20	80	20 (10)	160	10	160

Notes: MBC: minimum bactericidal concentration; MIC: minimum inhibitory concentration. Additional MIC values are presented in round parentheses when different results were obtained for distinct replications.
